# Differential Effects of Selective Inhibitors Targeting the PI3K/AKT/mTOR Pathway in Acute Lymphoblastic Leukemia 

**DOI:** 10.1371/journal.pone.0080070

**Published:** 2013-11-14

**Authors:** Susanne Badura, Tamara Tesanovic, Heike Pfeifer, Sylvia Wystub, Bart A. Nijmeijer, Marcus Liebermann, J. H. Frederik Falkenburg, Martin Ruthardt, Oliver G. Ottmann

**Affiliations:** 1 Department of Medicine, Hematology and Oncology, Johann Wolfgang Goethe University, Frankfurt, Germany; 2 Department of Hematology, Leiden University Medical Center, Leiden, The Netherlands; Westmead Millennium Institute, University of Sydney, Australia

## Abstract

**Purpose:**

Aberrant PI3K/AKT/mTOR signaling has been linked to oncogenesis and therapy resistance in various malignancies including leukemias. In Philadelphia chromosome (Ph) positive leukemias, activation of PI3K by dysregulated BCR-ABL tyrosine kinase (TK) contributes to the pathogenesis and development of resistance to ABL-TK inhibitors (TKI). The PI3K pathway thus is an attractive therapeutic target in BCR-ABL positive leukemias, but its role in BCR-ABL negative ALL is conjectural. Moreover, the functional contribution of individual components of the PI3K pathway in ALL has not been established.

**Experimental Design:**

We compared the activity of the ATP-competitive pan-PI3K inhibitor NVP-BKM120, the allosteric mTORC1 inhibitor RAD001, the ATP-competitive dual PI3K/mTORC1/C2 inhibitors NVP-BEZ235 and NVP-BGT226 and the combined mTORC1 and mTORC2 inhibitors Torin 1, PP242 and KU-0063794 using long-term cultures of ALL cells (ALL-LTC) from patients with B-precursor ALL that expressed the BCR-ABL or TEL-ABL oncoproteins or were BCR-ABL negative.

**Results:**

Dual PI3K/mTOR inhibitors profoundly inhibited growth and survival of ALL cells irrespective of their genetic subtype and their responsiveness to ABL-TKI. Combined suppression of PI3K, mTORC1 and mTORC2 displayed greater antileukemic activity than selective inhibitors of PI3K, mTORC1 or mTORC1 and mTORC2.

**Conclusions:**

Inhibition of the PI3K/mTOR pathway is a promising therapeutic approach in patients with ALL. Greater antileukemic activity of dual PI3K/mTORC1/C2 inhibitors appears to be due to the redundant function of PI3K and mTOR. Clinical trials examining dual PI3K/mTORC1/C2 inhibitors in patients with B-precursor ALL are warranted, and should not be restricted to particular genetic subtypes.

## Introduction

The Phosphatidylinositol 3-kinase (PI3K) signaling pathway plays an important role in many physiological functions, including cell cycle progression, differentiation, survival, apoptosis and protein synthesis [1,2]. Dysregulated PI3K signaling has been linked to oncogenesis and disease progression in a variety of solid tumors and hematologic malignancies and appears to enhance resistance to antineoplastic therapy, resulting in a poor prognosis [1–4]. Aberrant PI3K/AKT activation has been reported in 50% to 80% of acute myeloid leukemias (AML), up to 88% of acute T-lymphoblastic leukemias (ALL), and in chronic myeloid leukemia (CML) [5–7]. In CML, activation of the PI3K pathway has been linked to the BCR-ABL tyrosine kinase, the hallmark of CML which is also present in approximately 25% of adult ALL patients, coinciding with the presence of the Philadelphia (Ph) chromosome [3,8,9]. The prognosis of patients with Ph+ ALL remains poor and is limited by the development of secondary resistance to ABL-directed tyrosine kinase inhibitors (TKI), caused predominantly by BCR-ABL tyrosine kinase domain (TKD) mutations that prevent the TKI-induced inhibition of BCR-ABL activity [8,10–12]. This results in continued activation of multiple signaling pathways downstream of BCR-ABL, of which PI3K/AKT plays a pivotal role due to its widely accepted involvement in BCR-ABL mediated leukemogenesis [3,6,13,14]. Activation of the PI3K/AKT/mTOR pathway has also been shown to be involved in non-mutational resistance of BCR-ABL expressing cells to the ABL-directed tyrosine kinase inhibitor imatinib [15,16]. While these data make a compelling case for targeting the PI3K pathway as a therapeutic strategy for Ph+ ALL, its potential pathophysiologic role and value as a therapeutic target in BCR-ABL negative B-lineage ALL remain largely unexplored. 

Activation of PI3K leads to the phosphorylation of AKT on Thr308, which in turn induces activation of mammalian target of rapamycin (mTOR), a distal element of the PI3K/AKT/mTOR pathway [2,17,18]. mTOR is a serine/threonine kinase that encompasses two distinct complexes, mTORC1 and mTORC2, which differ structurally, in their substrate specificity and functionally [18,19]. mTORC1 is known to induce cell growth in response to nutrients and growth factors by regulating the translational regulators S6K1 and 4E-BP1, whereas mTORC2 mediates cell proliferation and survival by phosphorylating AKT on Ser473 to facilitate its full activation [17,18,20–24].

The relative contributions of the individual components of the PI3K/AKT/mTOR signaling pathway for proliferation and survival in the cellular context of ALL remain to be resolved. Combined inhibition of PI3K and the mTOR complexes 1 and 2 may have the advantage to inhibit feedback loops and may be more efficient than targeting PI3K and mTORC1 alone. 

A number of inhibitors of PI3K/AKT/mTOR signaling have been developed that selectively interfere with different components of this pathway, and thus differ in their biological effects. The allosteric mTORC1 inhibitors rapamycin and RAD001 display primarily antiproliferative effects *in vitro* and *in vivo*, with slowing of tumor growth and delayed progression, but are poor inducers of apoptosis. One reason for this appears to be the simultaneous activation of feedback loops in conjunction with inhibition of mTORC1, resulting in survival of cells [25–33]. 

As PI3K signaling is considered to be one of the decisive pathways for the transformation potential of BCR-ABL, and may play a role in causing imatinib-resistance, we investigated the antileukemic effects of PI3K, mTORC1 and combined PI3K, mTORC1 and mTORC2 inhibition in BCR-ABL/TEL-ABL positive and negative LTCs. 

We utilized several inhibitors that are currently in clinical testing, including the ATP-competitive selective pan-PI3K inhibitor NVP-BKM120, the dual PI3K and mTOR inhibitors NVP-BEZ235 and NVP-BGT226, and the allosteric mTORC1 inhibitor RAD001[34–37]. We examined the following issues related to the potential anti-leukemic activity of PI3K pathway inhibition: i) Susceptibility of BCR-ABL positive ALL resistant to ABL-directed TKI to PI3K pathway inhibitors ii) Comparison of TEL-ABL positive with BCR-ABL positive ALL cells in terms of the functional relevance of PI3K signaling, iii) Activity of PI3K pathway inhibitors against BCR-ABL negative ALL iv) Assessment of the relative contribution of the different components of the PI3K pathway to leukemia growth and survival, and v) utility of assessing the phosphorylation status of AKT, S6 and 4E-BP1 as biomarkers for responsiveness among different leukemic subtypes 

## Materials and Methods

### ALL-LTCs, cells and reagents

Initiation of a LTC from a BCR-ABL positive ALL patient harboring the T315I mutation was performed essentially as previously described [38]. Briefly, leukemic blasts that had been collected from the bone marrow of a patient following development of resistance to dasatinib were cryopreserved after Ficoll density centrifugation, thawed and placed into serum-free culture medium. Viable cells were maintained at a density ranging from 0.5 to 2x10^6^ cells/ml. After an initial lag phase, proliferation ensued resulting in prolonged expansion of cultured cells. For all functional assays described herein, only cells that had been passaged for less than 6 months were used. All other ALL-LTCs from patients with BCR-ABL/TEL-ABL positive and negative B-precursor ALL have been described previously [38,39]. The LTCs showed largely stable karyotypes and immune phenotypes after 6 months in culture when compared to the corresponding primary cells. None of these 6 BCR-ABL positive LTCs harbored mutations in the ABL tyrosine kinase domain. Jurkat cells were obtained from the German Collection of Microorganisms and Cell Cultures (DSMZ, Braunschweig, Germany) and maintained in RPMI-1640 medium supplemented with 10% fetal calf serum (FCS) (Invitrogen, Karlsruhe, Germany). Imatinib, nilotinib (BCR-ABL inhibitors), NVP-BKM120 (PI3K inhibitor), RAD001 (mTORC1 inhibitor), NVP-BGT226, and NVP-BEZ235 (PI3K/mTORC1/2 inhibitors) were kindly provided from Novartis, Basel, Switzerland. LY294002 and Wortmannin (PI3K inhibitors) were obtained from Sigma, Steinheim, Germany, the mTORC1/C2 inhibitors Torin 1, PP242 and KU-0063794 were purchased from Selleck Chemicals (Munich, Germany) and dasatinib (BCR-ABL inhibitor) was kindly provided from Bristol-Myers Squibb, Munich, Germany.

### Proliferation assay

Cell proliferation was assessed using the Cell Proliferation Kit II (XTT) (Roche, Mannheim, Germany) according to the manufacturer´s instructions. Cells were seeded at a concentration of 0.5x10^6^ cells/ml, and proliferation was measured on day 4. Cell division of the LTC KÖ was determined by trypan blue dye exclusion assay using a Countess® Cell Counter (Invitrogen). 

### Assay for cell death

Cell death was quantified using the Annexin-V-FLUOS Staining Kit (Roche, Mannheim, Germany) according to the manufacturer´s instructions. Cells were seeded at a concentration of 0.5x10^6^ cells/ml and cell death was measured on day 4. Cell death was defined as positive staining for Annexin-V and/or propidium iodide by flow cytometry. 

### Western blotting

Western blot analyses were performed using the NuPage Western blotting system (Invitrogen) according to the manufacturer´s instructions. The following antibodies were used: anti-phosphorylated AKT specific for the phosphorylated serine residue 473 (p-AKT-S473), anti-AKT (AKT), anti-phosphorylated S6 protein specific for the phosphorylated serine residues 235/236 (p-S6-S235/236) and 240/244 (p-S6-240/244) anti-S6 (S6), anti-phosphorylated 4E-BP1, specific for the phosphorylated threonine residues Thr37/46 (p-4EB-P1-Thr37/46), anti-4E-BP1 (4E-BP1) and anti-β-Actin (β-Actin). All antibodies were obtained from Cell Signaling Technology, Danvers, MA. Membrane blocking and antibody incubation were performed in 5% low-fat dry milk and 5% BSA, respectively, and the membranes were washed in Tris-buffered saline (TBS) (10mM Tris-HCl pH8, 150nM NaCl) containing 0.1% Tween-20 (TBST). The antibodies were diluted in 5% BSA and 5% low-fat dry milk, respectively. 

### Statistical analysis

Differences in response rate of BCR-ABL+/TEL-ABL+ versus BCR-ABL- LTCs towards the same concentration of single inhibitors were analyzed by Student's t-test. Statistical analyses were performed using the GraphPad Prism (GraphPad Software, San Diego, CA) software package. 

## Results

### Characterization of a newly established BCR-ABL positive human ALL-LTC harboring the T315I mutation

**Figure 1:**
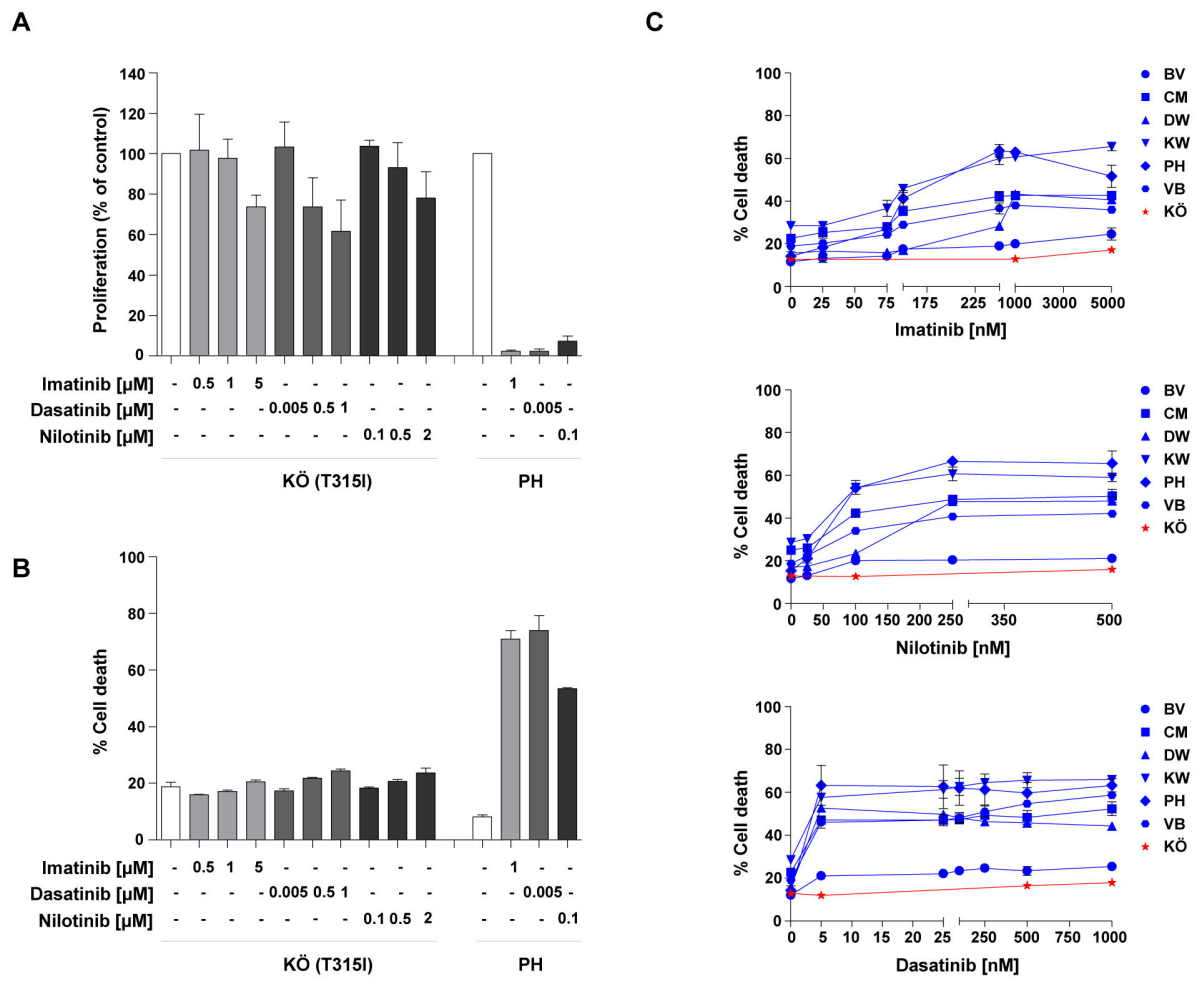
Effect of ABL-directed tyrosine kinase inhibitors on BCR-ABL+ ALL LTCs. Ph+ ALL cells with the T315I mutation showed no growth inhibition (A) or induction of cell death (B) in response to any of the TKI. The BCR-ABL+ ALL-LTC PH was used as a positive control (A and B). Response to ABL-directed TKI of 6 non-mutated BCR-ABL+ LTCs (BV, CM, DW, KW, PH and VB) and the LTC KÖ with the T315I mutation (C). Cell death was examined on day 4 of exposure to increasing concentrations of imatinib, dasatinib, and nilotinib. (A, B, C) Cell proliferation was assessed by XTT assay and induction of cell death was measured by Annexin-V/propidium iodide staining. The data shown represent the means + SD of 3 experimental replicates from one representative experiment out of 3 performed.

After the initial lag period, blast cells in the ALL-LTC KÖ had a doubling time of approximately 3 days. Surface marker expression demonstrated expression of CD19, CD79a, CD22, CD20, CD10, CD34, HLA-DR, TdT and CD13 (data not shown). Cytogenetic analysis at diagnosis revealed the following complex karyotype: 53,XX,del(1)(q25),+der(2)t(2;8)(q21;?),+6,der(9)t(1;9)(?;p22)t(9;22)(q34;q11),+14,+ der(18)t(1;18)(?;p11),+21+21,der(22)t(9;22)(q34;q11),der(22)t(9;22)(q34;q11). Mutation analysis of leukemic cells prior to and during culture demonstrated the presence of the BCR-ABL TKD gatekeeper mutation T315I. As expected, KÖ cells were unresponsive to imatinib, dasatinib and nilotinib when used at clinically achievable concentrations, known to inhibit proliferation and induce cell death in non-resistant BCR-ABL+ cells. The BCR-ABL+ LTC PH served as positive control (Figure 1A and 1B).

### BCR-ABL+ LTCs display variable responsiveness to TKI with concordance between imatinib, nilotinib and dasatinib

Functional *in vitro* analyses of mutational and non-mutational TKI-resistance of BCR-ABL+ ALL have relied on leukemic cell lines, given the lack of cell culture models using primary ALL cells. We employed the 6 ALL-LTC without TKD mutations described above to determine whether the cells differed in their innate responsiveness to the clinically available TKI imatinib, dasatinib and nilotinib, facilitating further studies of non-mutational resistance. Five of the 7 LTCs, demonstrated a dose–dependent but variable responsiveness to the TKI, one LTC (BV) was resistant despite no evidence of a TKD mutation (Figure 1C). The presence of the T315I mutation in KÖ cells was associated with resistance to all three TKI, as described above. Based on the antiproliferative and proapoptotic response (at 1µM imatinib, 250µM nilotinib and 25nM dasatinib, i.e. at plateau concentrations) we operationally classified the ALL-LTCs as highly sensitive (PH), intermediate sensitive (CM, DW, KW and VB), and resistant (KÖ and BV). While nilotinib and dasatinib where more potent than imatinib, the degree of TKI response was independent of the TKI used (Figure 1C).

Taken together, these ALL-LTCs recapitulate the different responses to TKI observed in patients, and represent the first suitable model for investigating mechanisms of non-mutational TKI resistance in BCR-ABL+ ALL, as well as for examining strategies to overcome this type of resistance.

### Impact of BCR-ABL and TEL-ABL activity on AKT and mTOR as downstream targets of PI3K

**Figure 2:**
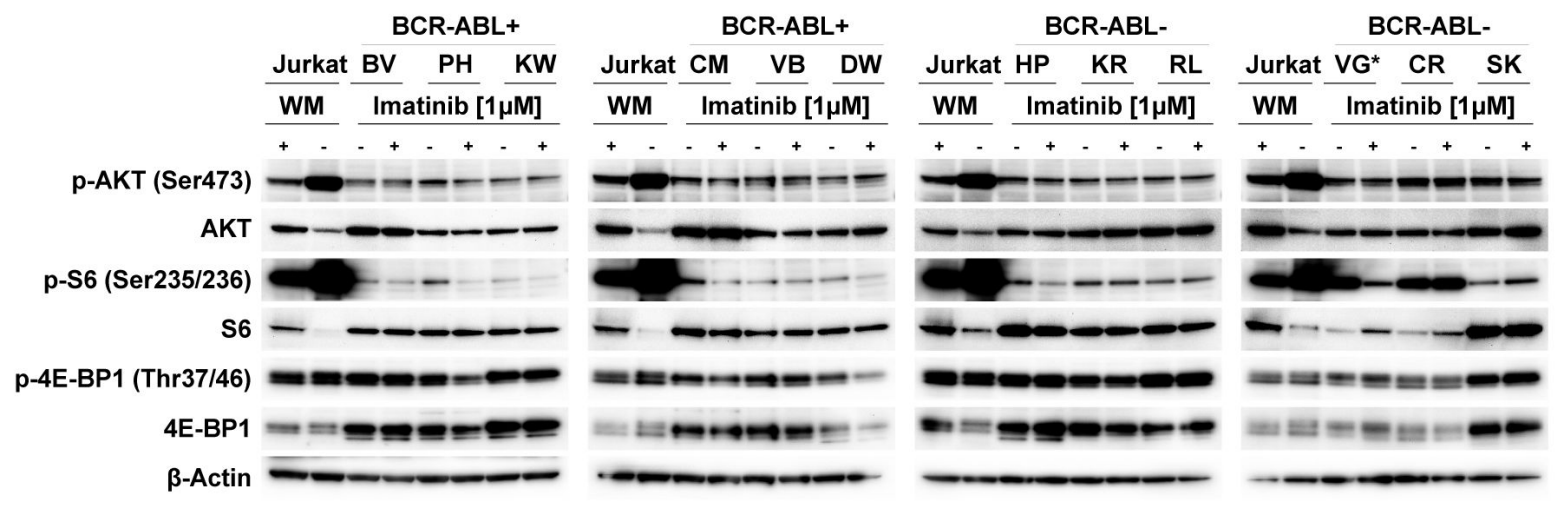
Impact of ABL-kinase inhibition on PI3K/AKT/mTOR signaling in BCR-ABL and TEL-ABL positive ALL LTCs. BCR-ABL+ (BV, PH, KW, CM, BV und DW), TEL-ABL+ (VG) and BCR-ABL- (HP, KR, RL, CR und SK) LTCs were treated with 1µM Imatinib for 20h. Lysates of these cells were used for the detection of phosphorylated and total AKT, S6 and 4E-BP1 by Western blotting. Because of the constitutively activated PI3K/AKT/mTOR pathway in Jurkat cells, lysates of untreated Jurkat cells were used as positive controls and that of cells treated for 2h with 1µM Wortmannin (WM), a PI3K inhibitor, were used as negative controls. β-Actin was used as loading control.

BCR-ABL activates numerous signaling pathways and thereby regulates cell proliferation and survival. The PI3K/AKT/mTOR pathway is considered to play a central role in BCR-ABL as well as in TEL-ABL induced leukemogenesis [3,6,40]. To examine whether inhibition of BCR-ABL and TEL-ABL kinase activity suppresses PI3K signaling, ALL-LTCs were exposed to imatinib at 1µM for 20h and phosphorylation levels of AKT, S6 protein and 4E-BP1 were determined. AKT phosphorylation at Ser473 was detected in all ALL-LTCs, irrespectively of the presence or absence of BCR-ABL or TEL-ABL, respectively (Figure 2). Unexpectedly, inhibition of BCR-ABL and TEL-ABL kinase activity did not result in dephosphorylation of AKT. To determine whether components of the PI3K pathway downstream of AKT were affected by inhibition of ABL, we analyzed the phosphorylation levels of the S6 protein and 4E-BP1. Phosphorylation of the S6 protein was more pronounced in untreated TEL-ABL+ LTC (VG) than in untreated BCR-ABL+ LTCs. In 3 of 7 LTCs with an ABL-rearrangement (2/6 BCR-ABL, 1/1 TEL-ABL) inhibition by imatinib resulted in dephosphorylation of the S6 protein, a well-established marker for the activity of mTORC1. In contrast, no dephosphorylation of 4E-BP1 was observed following exposure to imatinib in any of these cells (Figure 2). 

Thus, phosphorylation of AKT appears to be a common feature of B-precursor ALL and is not restricted to ALL cells with an ABL translocation. Moreover, the level of AKT phosphorylation does not depend on the tyrosine kinase activity of ABL, indicating that ABL-directed TKI exert their biologic effects through other components of the PI3K signaling pathway. Our observation that inhibition of BCR-ABL or TEL-ABL resulted in dephosphorylation of the S6 protein in only 3 of 7 cases (2/6 BCR-ABL, 1/1 TEL-ABL), and that the extent of imatinib-induced S6 dephosphorylation does not correlate with the sensitivity of the BCR-ABL+ ALL-LTCs to imatinib, point to an unexpected heterogeneity in mechanisms of TKI-induced inhibition. 

### The effects of PI3K inhibition are independent of BCR-ABL or TEL-ABL

The commonly accepted role of PI3K/AKT/mTOR as a major downstream signaling pathway of BCR-ABL and TEL-ABL suggested that cells harboring an ABL-translocation might be more sensitive to inhibition of this pathway than BCR-ABL negative cells. In addition, we were interested whether the variable sensitivity of the BCR-ABL+ LTCs to ABL-directed TKI was associated with a differential responsiveness to inhibition of the PI3K/AKT/mTOR pathway.

**Figure 3:**
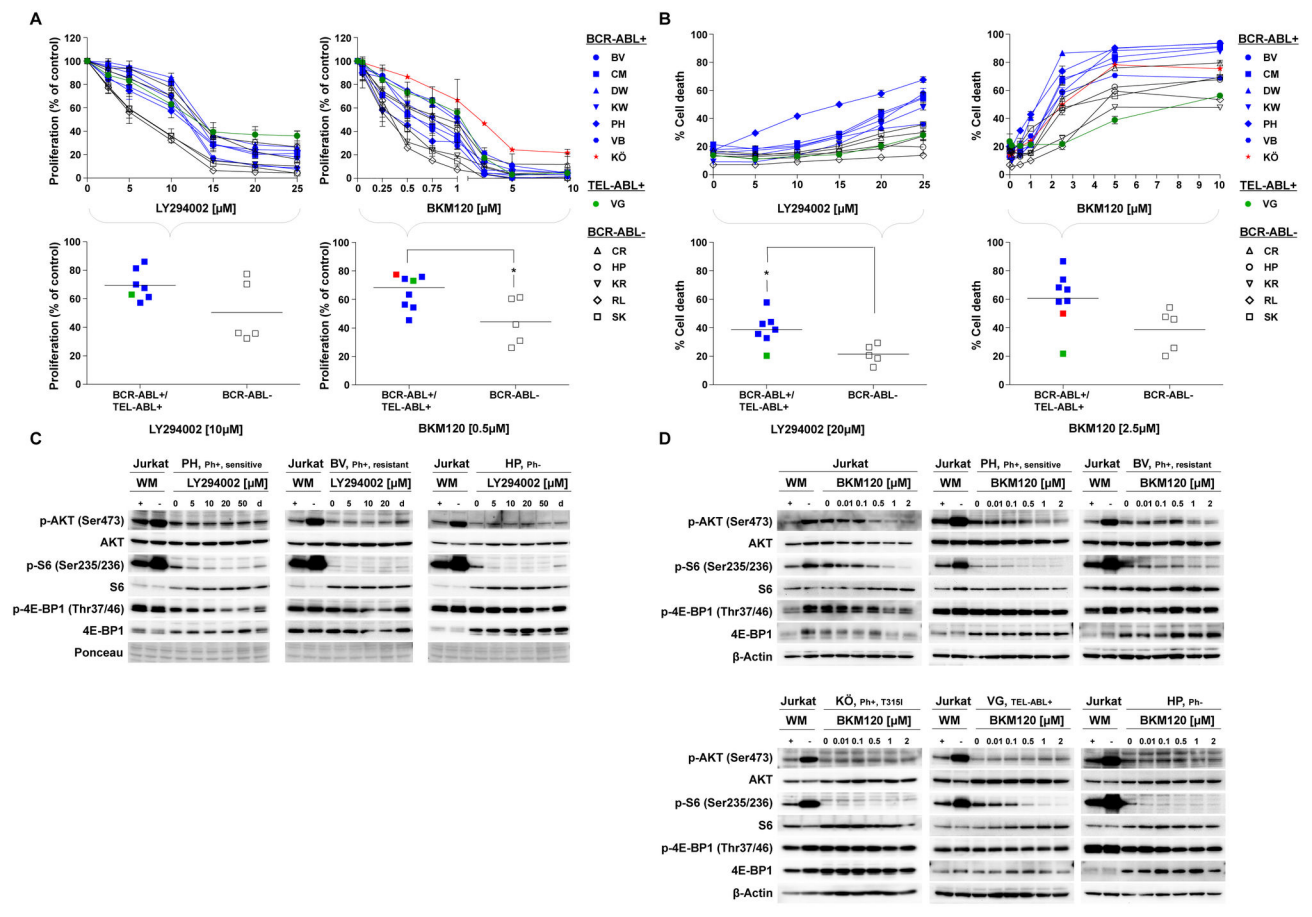
The impact of PI3K inhibition in B-ALL is independent of the presence of an ABL translocation. BCR-ABL+ (BV, PH, KW, CM, BV und DW), TEL-ABL+ (VG) and BCR-ABL- (HP, KR, RL, CR und SK) LTCs were exposed to increasing concentrations of the PI3K inhibitors LY294002 or NVP-BKM120. (A) Proliferation was measured after 4 days of drug treatment. Comparing the proliferation rate of the ABL-translocated cells (BCR-ABL+/TEL-ABL+) with the BCR-ABL- cells shows no difference in response to treatment with 10µM LY294002 (correspond approximately to the IC_50_). In contrast, exposure to 0.5µM NVP-BKM120 (correspond approximately to the IC_50_) resulted in a significantly stronger inhibition of proliferation of the BCR-ABL- cells (p=0.0221 (*)). (B) Cell death was measured after 4 days of drug treatment. The rate of cell death of the ABL-translocated cells (BCR-ABL+/TEL-ABL+) was significantly higher than of BCR-ABL negative ALL (p=0.013 (*)) after exposure of 20µM LY294002 (corresponding approximately to the IC_50_). Treatment with 2.5µM NVP-BKM120 showed no difference between ABL-translocated cells (BCR-ABL+/TEL-ABL+) and the BCR-ABL- cells in terms of cell death induction (A, B) Cell proliferation was assessed by using XTT assay, induction of cell death was measured by Annexin-V/propidium iodide staining. The data shown represent the means + SD of 3 experimental replicates from one representative experiment out of 3 performed. (C) BCR-ABL+ (PH and BV) and BCR-ABL- (HP) LTCs were treated with increasing concentrations of LY294002 for 2h. (D) BCR-ABL+ (PH, BV and KÖ), TEL-ABL+ (VG), BCR-ABL- (HP) and Jurkat cells were exposed to increasing concentrations of NVP-BKM120 for 2h. (C, D) Lysates of these cells were used for the detection of phosphorylated and total AKT, S6 and 4E-BP1 by Western blotting. Lysates of untreated Jurkat cells were used as positive controls and those of cells treated with the PI3K inhibitor Wortmannin (WM) for 2h at 1µM served as negative controls. Ponceau staining and β-Actin were used as loading control. d = DMSO control.

In all LTCs, the selective PI3K inhibitors NVP-BKM120 and LY294002 inhibited cell proliferation and induced cell death in a dose-dependent manner, although the sensitivity to PI3K inhibition varied (Figure 3A and 3B). Unexpectedly, the antiproliferative effect of PI3K inhibition was more pronounced in BCR-ABL negative ALL cells: At concentrations close to the IC_50_, LY294002 and NVP-BKM120 inhibited proliferation by a median of 30% in ALL with an ABL-translocation (BCR-ABL+/TEL-ABL+) and by 50% and 55% in BCR-ABL negative cells (p=0.022 for NVP-BKM120) (Figure 3A). 

Induction of cell death of ALL cells by both PI3K inhibitors was likewise dose-dependent. BCR-ABL/TEL-ABL+ ALL cells were more sensitive to induction of cell death than cells without an ABL-translocation, with median of 39% vs. 20% cell death in response to LY294002 (p=0.013) and 60% vs. 40% with NVP-BKM120 (Figure 3B). The antiproliferative and proapoptotic effects of NVP-BKM120 were more pronounced than those of LY294002, although the sensitivity of the individual ALL-LTCs was highly variable. In order to determine whether the heterogeneity of these antiproliferative and proapoptotic responses were associated with differential effects on PI3K signaling, we examined the phosphorylation levels of AKT, S6 and 4E-BP1, all of which are downstream of PI3K, in 5 ALL-LTCs representing different genetic subtypes of ALL. Jurkat cells have a constitutively activated PI3K pathway and were used as controls (Figure 3C and 3D). 

Unexpectedly, inhibition of PI3K by LY294002 and NVP-BKM120 was not associated with dephosphorylation of AKT at Ser473, a surrogate marker for inhibition of PI3K activity, in the majority of the ALL-LTCs, whereas AKT dephosphorylation was observed in the control Jurkat cells. To determine whether selective inhibition of PI3K had an effect on more distal components of this pathway, we examined the phosphorylation status of S6 and 4E-BP1, two targets of mTORC1 which are downstream of AKT. In the majority of ALL-LTCs and in the control Jurkat cells, S6 phosphorylation was reduced in dose-dependent manner in response to both LY294002 and NVP-BKM120, indicating that PI3K inhibition indeed inhibited mTORC1. In contrast, when ALL-LTCs were treated with these PI3K inhibitors, 4E-BP1 was not dephosphorylated (Figure 3C and 3D). 

Thus, in ALL-LTCs, the magnitude of the antiproliferative and proapoptotic effects of selective PI3K inhibition is independent of the presence of an ABL translocation. This is consistent with the observation that the effect of PI3K inhibition on the phosphorylation status of more distal components of the PI3K pathway was also independent of an ABL-translocation. Among BCR-ABL positive ALL-LTC, their sensitivity to ABL-directed TKI did not correlate with responsiveness to PI3K inhibition. The variable sensitivity of the LTCs to PI3K inhibition could not be attributed to differences in the phosphorylation of AKT, S6 protein and 4E-BP1.

### Inhibition of mTORC1 blocks cell proliferation without inducing cell death irrespectively of BCR-ABL/TEL-ABL status

**Figure 4:**
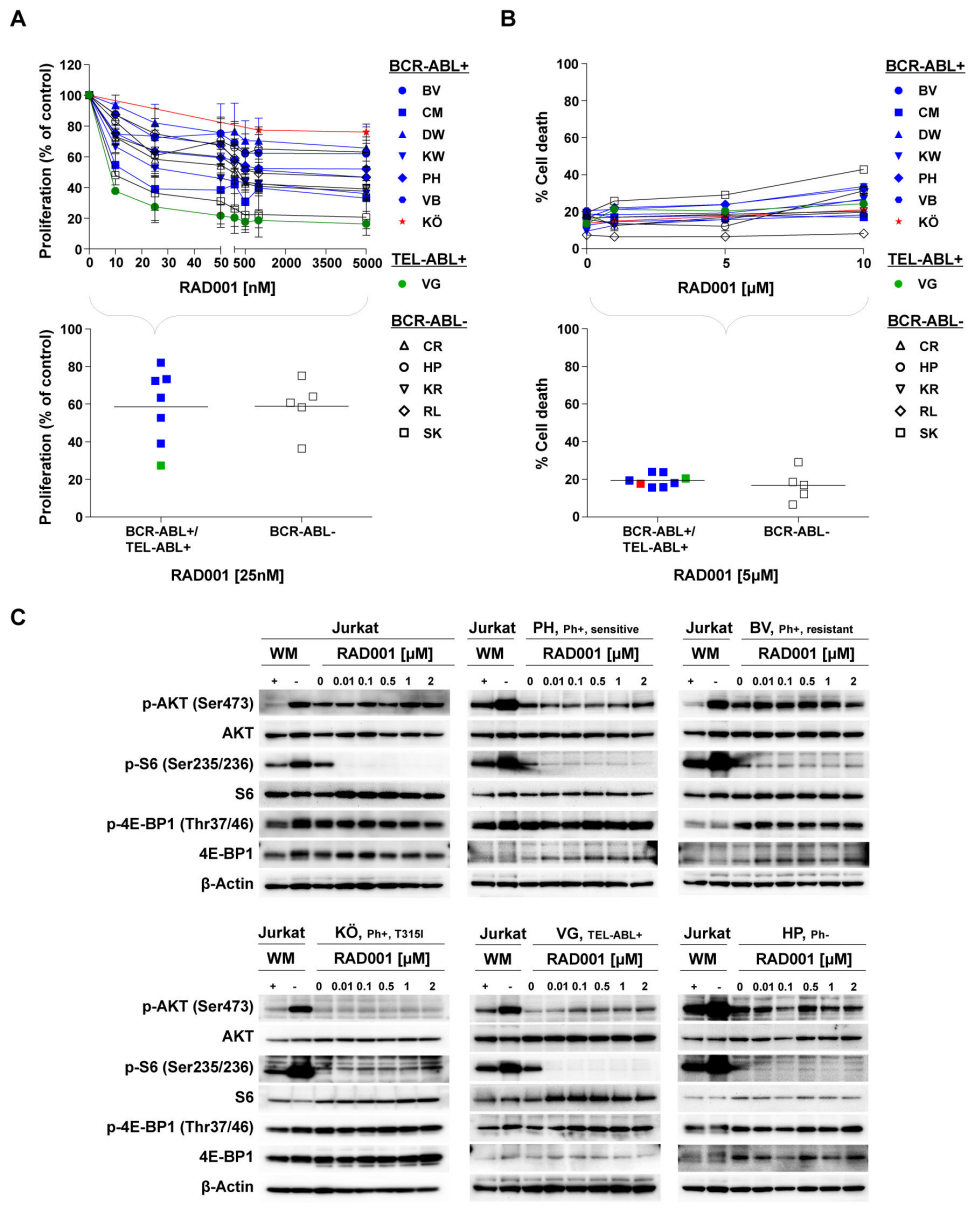
The impact of mTORC1 inhibition in B-ALL is independent of the presence of an ABL translocation. BCR-ABL+ (BV, PH, KW, CM, BV und DW), TEL-ABL+ (VG) and BCR-ABL- (HP, KR, RL, CR und SK) LTCs were exposed to increasing concentrations of the mTORC1 inhibitor RAD001. (A) Proliferation and (B) cell death were measured after 4 days of drug treatment. The (A) proliferation rate and (B) rate of cell death of the ABL-translocated cells (BCR-ABL+/TEL-ABL+) and the BCR-ABL- cells did not differ in their response to treatment with RAD001 at 25nM or 5µM, respectively (corresponding approximately to the IC_50_) values determined for growth inhibition and induction of apoptosis, respectively). (A, B) Cell proliferation was assessed by XTT assay, induction of apoptosis was measured by Annexin-V/propidium iodide staining. The data shown represent the means + SD of 3 experimental replicates from one representative experiment out of 3 performed. (C) BCR-ABL+ (PH, BV and KÖ), TEL-ABL+ (VG), BCR-ABL- (HP) and Jurkat cells were treated with increasing concentrations of RAD001 for 2h. Lysates of these cells were used for the detection of phosphorylated and total AKT, S6 and 4E-BP1 by Western blotting. Lysates of untreated Jurkat cells were used as positive controls and those of cells treated with the PI3K inhibitor Wortmannin (WM) for 2h at 1µM were used as negative controls. β-Actin was used as loading control.

The antiproliferative and proapoptotic effect of PI3K inhibition occurred in conjunction with dephosphorylation of the S6 protein, a downstream target of mTORC1. We therefore investigated whether selective inhibition of mTORC1 by RAD001 likewise resulted in suppression of proliferation and induction of cell death in ALL cells. RAD001 strongly inhibited phosphorylation of the S6 protein in the majority of ALL LTCs and Jurkat cells, but not of 4E-BP1 (Figure 4C). This was associated with a dose-dependent inhibition of cell proliferation ranging from 20%-70% at 25nM in the ALL-LTCs tested, with maximum inhibition of cell growth at 100nM (Figure 4A). There was no statistically significant difference between ALL cells with or without an ABL-translocation. In contrast, RAD001 did not induce cell death of ALL cells from any of the LTCs even at concentrations up to 10µM (Figure 4B). Thus, while selective inhibition of PI3K by NVP-BKM120 and of mTORC1 by RAD001 had the same differential effect on phosphorylation of S6 protein and 4E-BP1, they differed considerably in their ability to induce cell death. This suggests that in ALL, the effect of PI3K signaling on survival and cell death is not mediated solely by mTORC1, and that phosphorylation of the mTORC1 targets S6 protein and 4E-BP1 is differentially regulated. Notably, exposure of TEL-ABL+ cells (VG) to RAD001 was accompanied by a compensatory increase in AKT phosphorylation (Figure 4C), a finding consistent with activation of negative feedback loops as a consequence of mTORC1 inhibition. This effect was not observed in any of the other BCR-ABL positive or negative cells. 

Moreover, the different sensitivity of the individual ALL-LTCs to mTORC1 inhibition does not correlate with the phosphorylation pattern of the pathway components as determined by Western blotting. 

### Antiproliferative and proapoptotic activity of combined inhibition of PI3K, mTORC1 and mTORC2 is independent of an ABL-translocation

**Figure 5:**
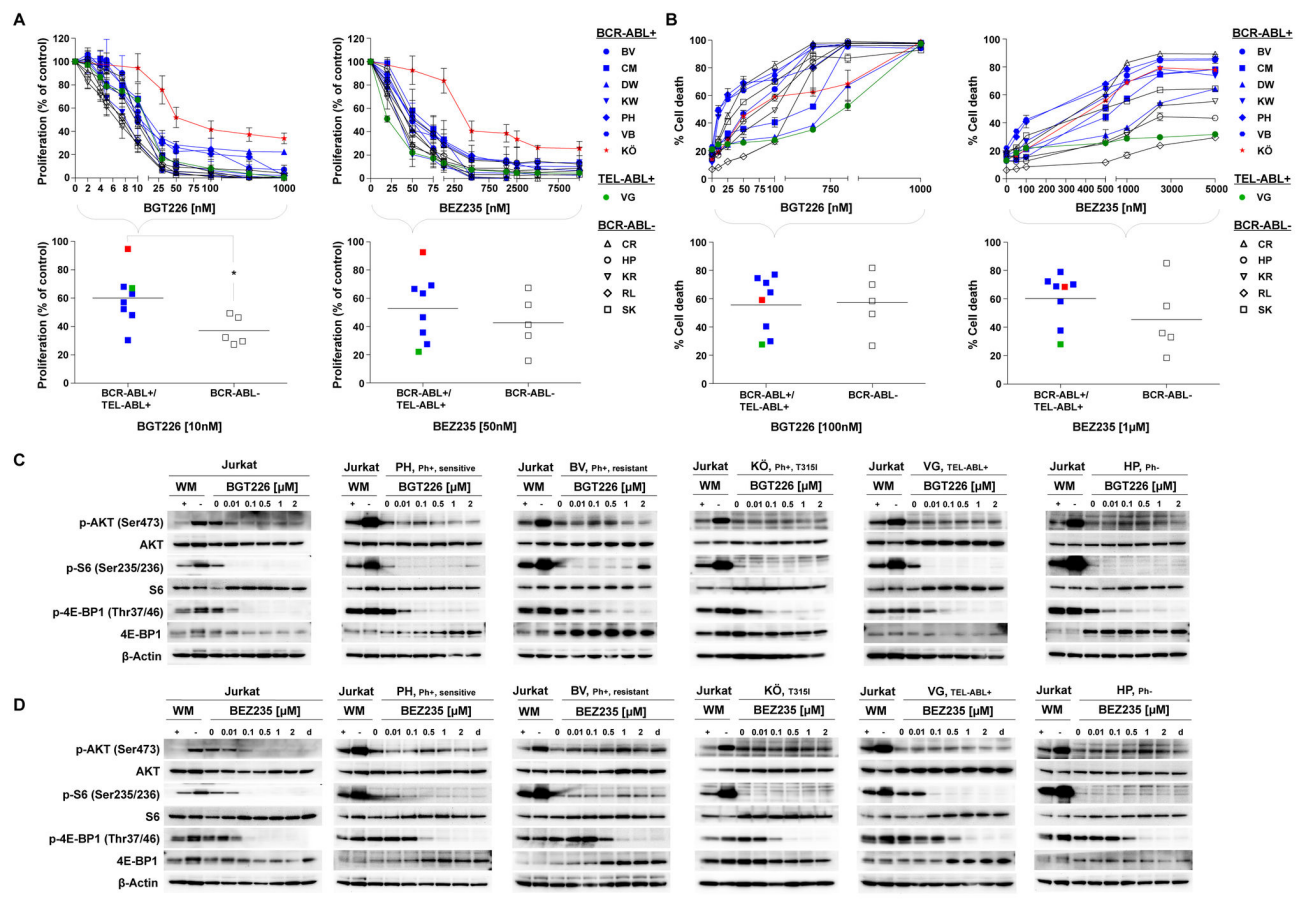
The impact of combined PI3K, mTORC1 and mTORC2 inhibition in B-ALL is independent of the presence of an ABL translocation. BCR-ABL+ (BV, PH, KW, CM, BV und DW), TEL-ABL+ (VG) and BCR-ABL- (HP, KR, RL, CR und SK) LTCs were exposed to increasing concentrations of the PI3K/mTORC1/C2 inhibitors NVP-BGT226 and NVP-BEZ235. (A) Proliferation was measured after 4 days of drug treatment. Inhibition of proliferation by 10nM NVP-BGT226 (corresponding approximately to the IC_50_) was more pronounced in the ABL-translocated cells (BCR-ABL+/TEL-ABL+) than in BCR-ABL- cells (p=0.0283 (*)). In contrast, treatment with 50nM NVP-BEZ235 (corresponding approximately to the IC_50_) showed no difference between BCR-ABL+/TEL-ABL+ and BCR-ABL- cells. (B) Apoptosis was measured after 4 days of drug exposure. The rate of cell death induced by NVP-BEZ235 or NVP-BGT226 was not significantly different in ABL-translocated cells (BCR-ABL+/TEL-ABL+) and BCR-ABL- cells (A, B) Cell proliferation was assessed by XTT assay, induction of cell death was measured by Annexin-V/propidium iodide staining. The data shown represent the means + SD of 3 experimental replicates from one representative experiment out of 3 performed. (C, D) BCR-ABL+ (PH, BV and KÖ), TEL-ABL+ (VG), BCR-ABL- (HP) and Jurkat cells were treated with increasing concentrations of (C) NVP-BGT226 or (D) NVP-BEZ235 for 2h. Lysates of these cells were used for the detection of phosphorylated and total AKT, S6 and 4E-BP1 by Western blotting. Lysates of untreated Jurkat cells were used as positive controls and those of cells treated for 2h with 1µM Wortmannin (WM) served as negative controls. β-Actin was used as loading control. d = DMSO control.

Whereas both RAD001 and NVP-BKM120 resulted in comparable dephosphorylation of S6 protein, cell death was induced only by NVP-BKM120. This prompted us to explore whether induction of cell death required inhibition of both mTORC2 and mTORC1. mTORC2 is known to induce cell proliferation by providing a feedback loop for AKT activation, which results in the phosphorylation of AKT at Ser473. The dual PI3K/mTORC1/C2 inhibitors NVP-BGT226 and NVP-BEZ235 dose-dependently inhibited proliferation and induced cell death in all ALL-LTCs (Figure 5A and 5B). Based on their IC_50_ values within the nanomolar range, both NVP-BGT226 and NVP-BEZ235 were more potent in terms of growth inhibition and induction of cell death than the selective inhibitors of PI3K and mTORC1 respectively (Figure 5A and 5B). We observed same effects with the mTORC1/C2 inhibitors Torin 1, PP242 and KU-0063794 however, IC_50_ values were in high nanomolar range for inhibition of proliferation and micromolar concentrations were needed for induction of cell death (Figures S1A and S1B).

Treatment with NVP-BGT226 and NVP-BEZ235 at concentrations close to the IC_50_ (NVP-BGT226 [10nM], NVP-BEZ235 [50nM]), inhibited proliferation of BCR-ABL negative LTCs more potently than that of BCR-ABL/TEL-ABL positive cells, although this was significant only for NVP-BGT226 (p=0.0283 10nM) (Figure 5A). No differences were detected for the mTORC1/C2 inhibitors Torin 1, PP242 and KU-0063794 at concentrations close to the IC_50_ (Figures S1A and S1B). Inhibition of PI3K/mTORC1/C2 by NVP-BGT226 or NVP-BEZ235 induced cell death in a dose-dependent manner in all LTCs independently of the presence of BCR-ABL/TEL-ABL translocation with median rates of cell death of 60% and 45%, respectively (Figure 5B). The same is true for the mTORC1/C2 inhibitors Torin 1 and PP242 except for the mTORC1/C2 inhibitor KU-0063794, which showed a significant greater induction of cell death in BCR-ABL/TEL-ABL positive cells compared to negative cells (p=0.0209 5µM) (Figures S1A and S1B).

To determine why dual inhibitors targeting PI3K/mTORC1/C2 more potently suppressed cell proliferation and induced cell death than the selective inhibitors of PI3K and mTORC1, respectively, we analyzed the phosphorylation level of AKT, S6 and 4E-BP1 in ALL-LTCs following to the different inhibitors (Figure 5C and 5D). 

In contrast to their effects on Jurkat cells, used as a positive control, the dual inhibitors NVP-BGT226 and NVP-BEZ235, failed to dephosphorylate AKT in ALL-LTCs, similar to the selective PI3K and mTORC1 inhibition. S6 protein, downstream of AKT, was dephosphorylated following exposure to both dual (NVP-BGT226, NVP-BEZ235) and selective inhibitors (NVP-BKM120, RAD001) in the majority of ALL cells examined. In contrast, dephosphorylation of 4E-BP1, likewise downstream of AKT, was observed in response to ATP-competitive PI3K/mTOR and mTORC1/C2 inhibitors (Figure S2) but not to selective inhibitors of PI3K and the allosteric mTORC1 inhibitor RAD001. This differential response was noted in both Jurkat and ALL-LTC and was also independent of the ABL-translocation status. The dose-response of 4E-BP1 dephosphorylation revealed greater potency of NVP-BGT226 compared to NVP-BEZ235 and was associated with a greater proapoptotic effect of NVP-BGT226.

Together, these data suggest that inhibition of both mTORC1 and mTORC2 by ATP-competitive inhibitors contributes to the antiproliferative and proapoptotic effects of the dual inhibitors. At inhibitor concentrations that induce cell death and dephosphorylation of 4EBP1, Ser473 AKT does not appear to be involved, suggesting a role of other PI3K-related kinases such as SGK [41].

### Pronounced antiproliferative and proapoptotic activity of dual PI3K/mTORC1/mTORC2 inhibitors in ALL

**Figure 6:**
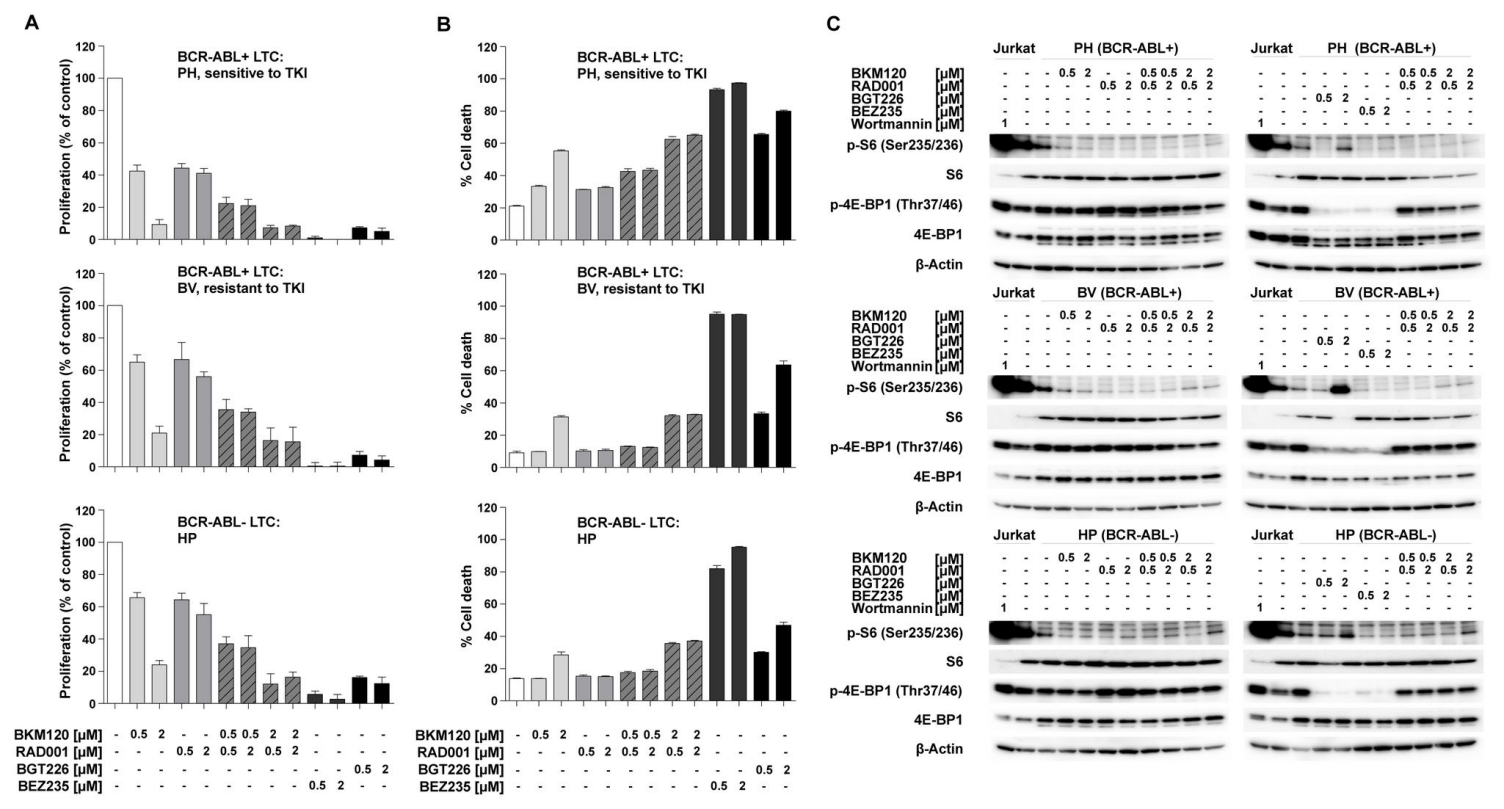
The impact of additional inhibition of mTORC2 on combined PI3K and mTORC1 inhibition in B-ALL is independent of the presence of an ABL translocation. BCR-ABL+ (PH and BV) and BCR-ABL- (HP) cells were treated with 0.5µM or 2µM NVP-BKM120 (PI3K inhibitor), 0.5µM or 2µM RAD001 (mTORC1 inhibitor) alone or in combination. For combined PI3K/mTORC1/C2 inhibition, cells were treated with 0.5µM or 2µM of NVP-BGT226 or NVP-BEZ235. (A) Proliferation and (B) cell death were measured after 4 days of drug treatment. (A, B) Cell proliferation was assessed by XTT assay, induction of cell death was measured by Annexin-V/propidium iodide staining. The data shown represent the means + SD of 3 experimental replicates from one representative experiment out of 3 performed. (C) Lysates were prepared after 2h of drug treatment for the detection of phosphorylated and total AKT, S6 and 4E-BP1 by Western blotting. Lysates of untreated Jurkat cells were used as positive controls, those of cells treated for 2h with 1µM Wortmannin (WM) were used as negative controls. β-Actin was used as loading control.

The greater antiproliferative and proapoptotic activity of dual (ATP-competitive) inhibitors of PI3K and mTOR, in conjunction with the exclusively antiproliferative effects of allosteric mTORC1 inhibition, raised the possibility of a contributory role of mTORC2. To determine whether mTORC2 inhibition indeed contributes to the antileukemic effect of the dual inhibitors NVP-BGT226 and NVP-BEZ235, we compared the antileukemic activity of these compounds with that of the selective inhibitors alone and in combination. As shown in Figure 6A, inhibition of PI3K by the ATP-competitive inhibitor NVP-BKM120 in conjunction with the allosteric mTORC1 inhibitor RAD001 has a greater antiproliferative effect than treatment with the individual inhibitors alone. However, concurrent inhibition of PI3K and mTORC1 had a lesser antiproliferative effect than the combined inhibition of PI3K, mTORC1 and mTORC2 achieved with the dual inhibitors NVP-BGT226 and NVP-BEZ235, respectively. Cell death was not enhanced by combining the selective inhibitors, whereas simultaneous inhibition of PI3K, mTORC1 and mTORC2 by ATP competitive inhibitors considerably enhanced cell death, most pronounced for NVP-BGT226 (Figure 6B).

To analyze whether inhibition of mTORC2 affects the phosphorylation level of the S6 protein and 4E-BP1, we performed western blot analysis after exposure of the different drug combinations described above. As shown in Figure 6C, individual as well as combined PI3K and mTORC1 inhibition and combined PI3K, mTORC1 and mTORC2 inhibition results in the dephosphorylation of the S6 protein, as expected. In contrast, allosteric inhibition of mTORC1, alone or combined with a selective ATP-competitive inhibitor of PI3K had no impact on 4E-BP1 phosphorylation, whereas the simultaneous inhibition of mTORC1 and C2 or of PI3K, mTORC1 and mTORC2 by ATP-competitive inhibitors resulted in a dephosphorylation of 4EBP1 (Figure 6C and Figure S2). 

Taken together, these data indicate that the roles of mTORC1 and mTORC2 in regulating proliferation and survival of B-ALL cells are independent of an ABL-translocation. Furthermore, these data are consistent with a contribution of mTORC1 and/or mTORC2 to regulation of 4E-BP1 phosphorylation. 

## Discussion

Functional studies involving primary ALL cells are hampered by the well-known inability to maintain human ALL blasts in short-term culture and the high initial rate of cell death. We have previously reported a unique cell culture system enabling long-term culture of leukemic lymphoblasts obtained from patients with ALL. These cultures represent different genetically defined ALL subtypes, i.e. leukemias harboring the BCR-ABL, TEL-ABL or E2A-PBX1 translocation, or no recurring genetic abnormality [38,39]. Our evaluation of the role of the PI3K pathway in leukemic cell growth and survival focused initially on BCR-ABL expressing leukemias, as PI3K signaling has been strongly implicated in malignant transformation and development of TKI-resistance in Ph+ ALL [3,6,14–16]. Effective suppression of PI3K signaling utilizing two potent, dual specificity ATP-competitive compounds, both of which are pan-PI3K inhibitors and additionally block the mTOR complexes C1 and C2 downstream of PI3K (NVP-BGT226 and NVP-BEZ235), potently inhibits proliferation and induces cell death in BCR-ABL positive and in TEL-ABL positive ALL at nanomolar concentrations. The magnitude of the antileukemic effects did not differ in ALL cells that displayed high or intermediate sensitivity to the ABL-directed kinase inhibitors imatinib, nilotinib and dasatinib, or were resistant either due to the T315I TKD mutation (in KÖ cells) or non-mutational resistance (in BV cells). In conjunction with published data showing that blockage of the PI3K pathway with the dual inhibitor NVP-BEZ235 does not appreciably affect survival, clonogenic growth and differentiation of normal CD34 positive cells [42], the profound antileukemic activity against Ph+ ALL indicates a pivotal role of the PI3K pathway in our ALL cells. This is consistent with the generally accepted activation of PI3K by the BCR-ABL oncoprotein, but does not exclude its involvement in BCR-ABL negative ALL as well. In fact, aberrant activation of the PI3K pathway has been shown to be one of the most frequent perturbations of signaling pathways in malignancies, including leukemias [5–7]. Our results with BCR-ABL/TEL-ABL negative ALL cells demonstrate for the first time that the antiproliferative and proapoptotic effects of the dual inhibitors NVP-BGT226 and NVP-BEZ235 are comparable to those in ABL-translocated ALL. The mechanism by which the PI3K pathway is activated in ALL cells is presently unclear. Possible mechanisms include constitutive activation of upstream signaling pathways, e.g. by BCR-ABL or inactivation of PTEN as shown in T-ALL lines, activating mutations of NOTCH1, RAS, or of the PI3K itself as in B-lymphomas [43–45]. Activating mutations of PI3K and AKT occur frequent in solid tumors but are rarely observed in leukemias [46–49]. Nevertheless, we sequenced the hot-spot regions of *PIK3CA* and screened for the E17K mutation in the *AKT1* gene, confirming that no mutations are present in any of the 13 ALL-LTCs (data not shown). Mutations or epigenetic silencing of PTEN occur in many tumor types including Ph+ ALL [50], resulting in enhanced phosphorylation of AKT by increasing cellular levels of PIP_3_. We found no evidence of decreased PTEN expression in our ALL cells, indicating that diminished PTEN activity is not the cause of AKT activation. 

Our results raise the question which of the individual components of the PI3K/AKT/mTOR pathway are most relevant for supporting leukemic cell growth and thus are the most attractive targets for therapeutic intervention. To dissect the relative contributions of PI3K and the mTORC1 and mTORC2 complexes, we exposed ALL-LTCs to the allosteric mTORC1 inhibitor RAD001 and the selective panPI3K inhibitor NVP-BKM120, both of which are being evaluated for anti-neoplastic activity in clinical trials. Blocking only mTORC1 by RAD001 failed to induce cell death in any of the ALL cells, and had a moderate antiproliferative effect, which was most conspicuous in the TEL-ABL expressing ALL cells. These findings are in line with numerous preclinical studies of various disease entities, which demonstrate that RAD001 has primarily antiproliferative effects [25,27,32]. This suggests that allosteric inhibition of mTORC1 alone by RAD001 is unlikely to be an effective antileukemic approach in ALL. Suppression of PI3K activity by the selective inhibitor NVP-BKM120 had both antiproliferative and significant cell death-inducing activity in the ALL-LTC, irrespective of their underlying genetic abnormalities. The lack of proapoptotic activity of RAD001 or known rapalogs may be attributable to the activation of a feedback loop that leads to enhanced phosphorylation of PI3K and subsequent activation of AKT, which in turn counteracts the growth inhibitory effect of mTORC1 inhibition [27–30]. The impact of this feedback loop on PI3K activity should be abrogated by blocking this pathway proximally, e.g. by the selective PI3K inhibitor NVP-BKM120. Indication of such a feedback loop in our ALL cells is provided most visibly in experiments using the TEL-ABL positive ALL-LTC VG, in which exposure to RAD001 resulted in a dose-dependent augmentation of AKT phosphorylation. Accordingly, combining inhibitors that act both distally (e.g. RAD001) and proximally (e.g. NVP-BKM120) is a rational approach towards maximizing the antileukemic efficacy of PI3K pathway inhibition. When we combined these two compounds, the antiproliferative activity was profoundly enhanced in comparison with the individual inhibitors, but we observed no induction of cell death. We therefore postulate that the poor proapoptotic activity of NVP-BKM120 and RAD001 in nanomolar concentrations could be due to their inability to block mTORC1 and mTORC2. Another potentially contributing factor is the limited suppressive effect of allosteric inhibitors on mTORC1, resulting in compensatory activation of feedback loops that may not be seen in the presence of ATP-competitive inhibitors of mTORC1 [51]. However, no clear evidence for activation of these well described feedback loops was noted in our experiments, based on the absence of increased AKT phosphorylation. While the ribosomal protein S6 is a S6K1 substrate and its phosphorylation is used as a biomarker of mTORC1 activity, the regulation of 4E-BP1 is controversial [17,52]. We demonstrate dephosphorylation of 4E-BP1 in response to ATP-competitive inhibitors of mTORC1 and C2 but not to allosteric mTORC1 inhibition or inhibition of PI3K. These data are not compatible with a recently published model which proposes that 4E-BP1 is under the direct control of PI3K [52]. While our data implicate mTOR as an important mediator of 4E-BP1 phosphorylation, we can not discriminate between the relative contributions of mTORC1 and mTORC2 because of the lack of selective, ATP-competitive inhibitors of these two mTOR components. Thus, it is possible that both mTORC1 and mTORC2 phosphorylate 4E-BP1 so that inhibition of either one would not dephosphorylate 4E-BP1 because of compensatory signaling by the other. These divergent findings may reflect the different cell context in the various studies, and support a model in which 4E-BP1 is regulated by both mTORC1 and mTORC2 in the setting of B-lineage acute lymphoblastic leukemia. In conjunction with the biologic data presented above, inhibition of leukemic cell proliferation and induction of cell death was by far most pronounced with the dual inhibitors NVP-BEZ235 and NVP-BGT226 that resulted in 4E-BP1 dephosphorylation. This is consistent with their ability to inhibit mTORC2 in addition to mTORC1 and PI3K, with the caveat that results based on pharmacologic rather than genetic inhibition may be influenced by off-target effects of the inhibitors used. 

We did not specifically examine and compare the impact of selective AKT inhibition on leukemic cell growth and cell death. By Western blotting, AKT showed only a minimal degree of phosphorylation at position Ser473 in the ALL-LTC compared to the Jurkat cell line. This is similar to published data showing weak AKT phosphorylation in p190^BCR-ABL^ transformed murine pro/pre-B cells, despite these cell´s dependency on PI3K signaling [53]. More importantly however, we observed no consistent dephosphorylation of AKT following inhibition of either BCR-ABL or PI3K signaling, raising the possibility that other targets within the PI3K signaling pathway may be more important than AKT. In numerous cancer cell lines, oncogenic PI3K activation has indeed recently been shown to be mediated not by AKT but by SGK3, another kinase that activates mTORC1 [41], suggesting that AKT itself may be only one of several relevant targets for antileukemic interventions targeting the PI3K pathway. However, Levy et al. [54] recently reported that the selective pan-AKT inhibitor GSK690693 possessed potent antiproliferative activity in 12 of 15 B-lineage ALL cell lines, including two that were BCR-ABL positive. Cell death was induced in all 3 T-ALL cells tested and in one BCR-ABL negative ALL cell line. These observations argue for a significant contributory role for AKT signaling in ALL growth, and lend support to our findings that PI3K signaling is a relevant antileukemic target not only in BCR-ABL positive leukemias, but also in other subtypes of ALL. As a caveat, comparison of the biologic effects of the various pharmacologic inhibitors needs to consider that off-target effects may contribute to the relative potencies of the agents used and may be confounding factors when interpreting the interactions between different agents. For example, a recent publication by Shortt J et al. [55] demonstrates that BEZ235 also inhibits the PI3K-related kinases ATM and DNA-PK at nanomolar concentrations. Thus, it is possible that the anti-apoptotic activity of BEZ235, and possibly of BGT266, is not exclusively due to inhibition of canonical PI3K signaling but to effects on other non-mTOR, PI3K- related kinases.

In conclusion, simultaneous inhibition of PI3K, mTORC1 and mTORC2 by the dual inhibitors NVP-BEZ235 and NVP-BGT226 exerts profound antileukemic activity against a broad spectrum of B-precursor ALL, irrespective of genetic subtype and – in the case of BCR-ABL positive ALL – their degree of responsiveness or resistance to clinically established ABL-kinase inhibitors. Combined inhibition of PI3K, mTORC1 and mTORC2 enhances the induction of cell death in a subset of these leukemias. In the cellular context of ALL, dephosphorylation of 4E-BP1 is observed in response to inhibition of both mTORC1 and mTORC2, but is not necessarily associated with induction of cell death. Our data provide a strong preclinical rationale for clinical studies exploring these compounds as treatment for ALL, but suggest that 4E-BP1 or S6 phosphorylation may not be robust biomarkers in clinical trials of PI3K pathway inhibitors in ALL. 

## Supporting Information

Figure S1 **The impact of combined mTORC1 and mTORC2 inhibition in B-ALL is independent of the presence of an ABL translocation.** BCR-ABL+ (BV, PH, KW, CM, BV und DW), TEL-ABL+ (VG) and BCR-ABL- (HP, KR, RL, CR und SK) LTCs were exposed to increasing concentrations of the mTORC1/C2 inhibitors KU-0063794, PP242 und Torin 1. (A) Proliferation was measured after 4 days of drug treatment. The proliferation rate of the ABL-translocated cells (BCR-ABL+/TEL-ABL+) and the BCR-ABL- cells did not differ in their response to treatment with KU-0063794, PP242 and Torin 1 at 1µM or 0.1µM, respectively (corresponding approximately to the IC_50_).
(B) Cell death was measured after 4 days of drug treatment. The rate of cell death of the ABL-translocated cells (BCR-ABL+/TEL-ABL+) was significantly higher than of BCR-ABL negative ALL (p=0.0209 (*)) after exposure of 5µM KU-0063794 (corresponding approximately to the IC_50_). Treatment with 5µM PP242 or 0.1µM Torin 1 showed no difference between ABL-translocated cells (BCR-ABL+/TEL-ABL+) and the BCR-ABL- cells in terms of cell death induction. (A, B) Cell proliferation was assessed by XTT assay, induction of cell death was measured by Annexin-V/propidium iodide staining. The data shown represent the means + SD of 3 experimental replicates from one representative experiment out of 2 performed.
(TIF)

Figure S2 **The impact of combined mTORC1 and mTORC2 inhibition in B-ALL on AKT, S6 and 4E-BP1 phosphorylation.** BCR-ABL+ (PH, BV) and Jurkat cells were treated with increasing concentrations of KU-0063794, PP242, Torin 1 for 2h. Lysates of these cells were used for the detection of phosphorylated and total AKT, S6 and 4E-BP1 by Western blotting. Lysates of untreated Jurkat cells were used as positive controls and those of cells treated for 2h with 1µM Wortmannin (WM) served as negative controls. β-Actin was used as loading control. d = DMSO control.
(TIF)
