# Supplementary figures and images for: Differential Effects of Selective Inhibitors Targeting the PI3K/AKT/mTOR Pathway in Acute Lymphoblastic Leukemia
Source: PLoS One. 2013 Nov 14;8(11):e80070. doi: 10.1371/journal.pone.0080070 (PMC3828226; doi:10.1371/journal.pone.0080070)

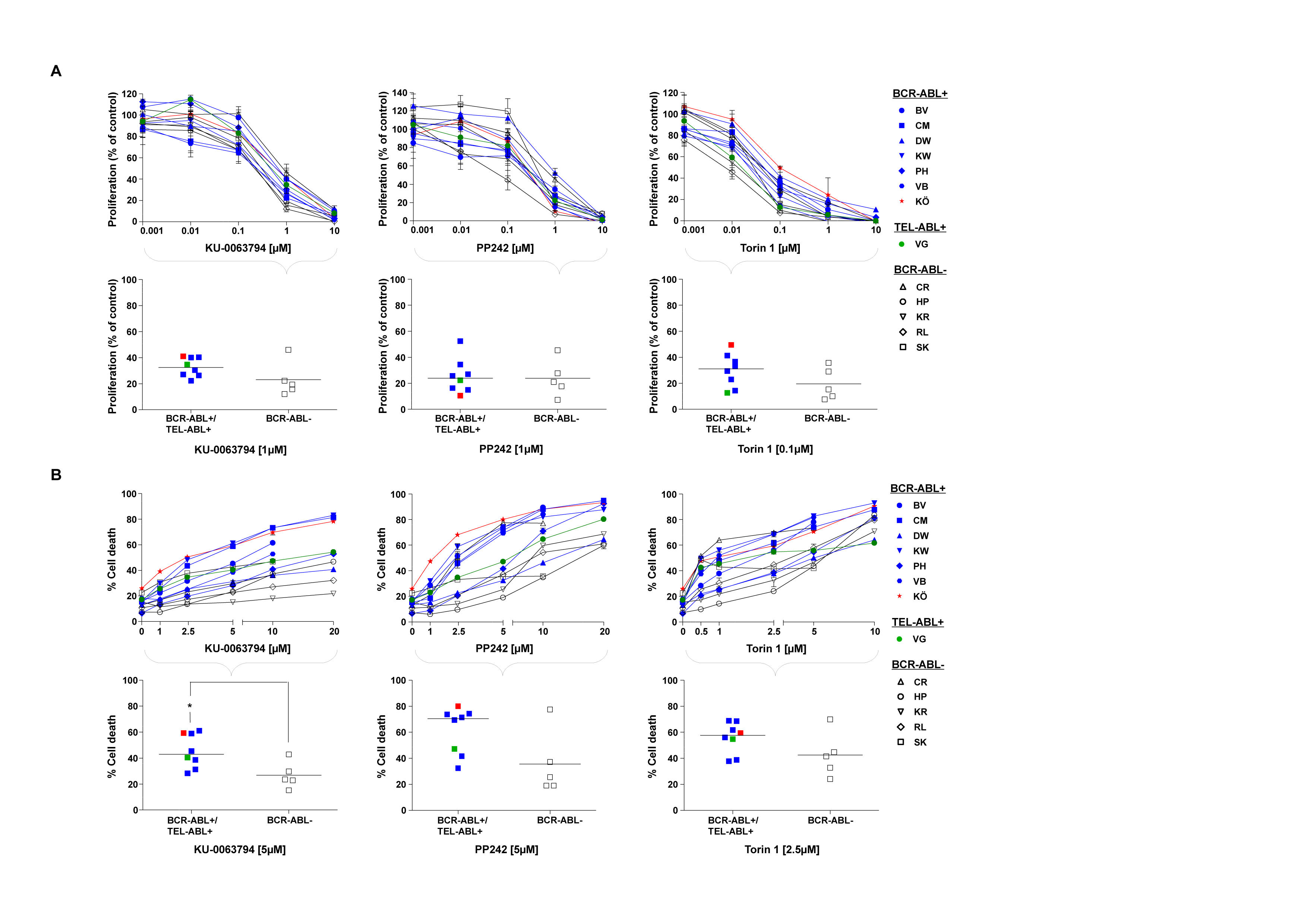

Supplement: Figure S1 — The impact of combined mTORC1 and mTORC2 inhibition in B-ALL is independent of the presence of an ABL translocation. BCR-ABL+ (BV, PH, KW, CM, BV und DW), TEL-ABL+ (VG) and BCR-ABL- (HP, KR, RL, CR und SK) LTCs were exposed to increasing concentrations of the mTORC1/C2 inhibitors KU-0063794, PP242 und Torin 1. (A) Proliferation was measured after 4 days of drug treatment. The proliferation rate of the ABL-translocated cells (BCR-ABL+/TEL-ABL+) and the BCR-ABL- cells did not differ in their response to treatment with KU-0063794, PP242 and Torin 1 at 1µM or 0.1µM, respectively (corresponding approximately to the IC50). (B) Cell death was measured after 4 days of drug treatment. The rate of cell death of the ABL-translocated cells (BCR-ABL+/TEL-ABL+) was significantly higher than of BCR-ABL negative ALL (p=0.0209 (*)) after exposure of 5µM KU-0063794 (corresponding approximately to the IC50). Treatment with 5µM PP242 or 0.1µM Torin 1 showed no difference between ABL-translocated cells (BCR-ABL+/TEL-ABL+) and the BCR-ABL- cells in terms of cell death induction. (A, B) Cell proliferation was assessed by XTT assay, induction of cell death was measured by Annexin-V/propidium iodide staining. The data shown represent the means + SD of 3 experimental replicates from one representative experiment out of 2 performed. (TIF) [file pone.0080070.s001.tif]

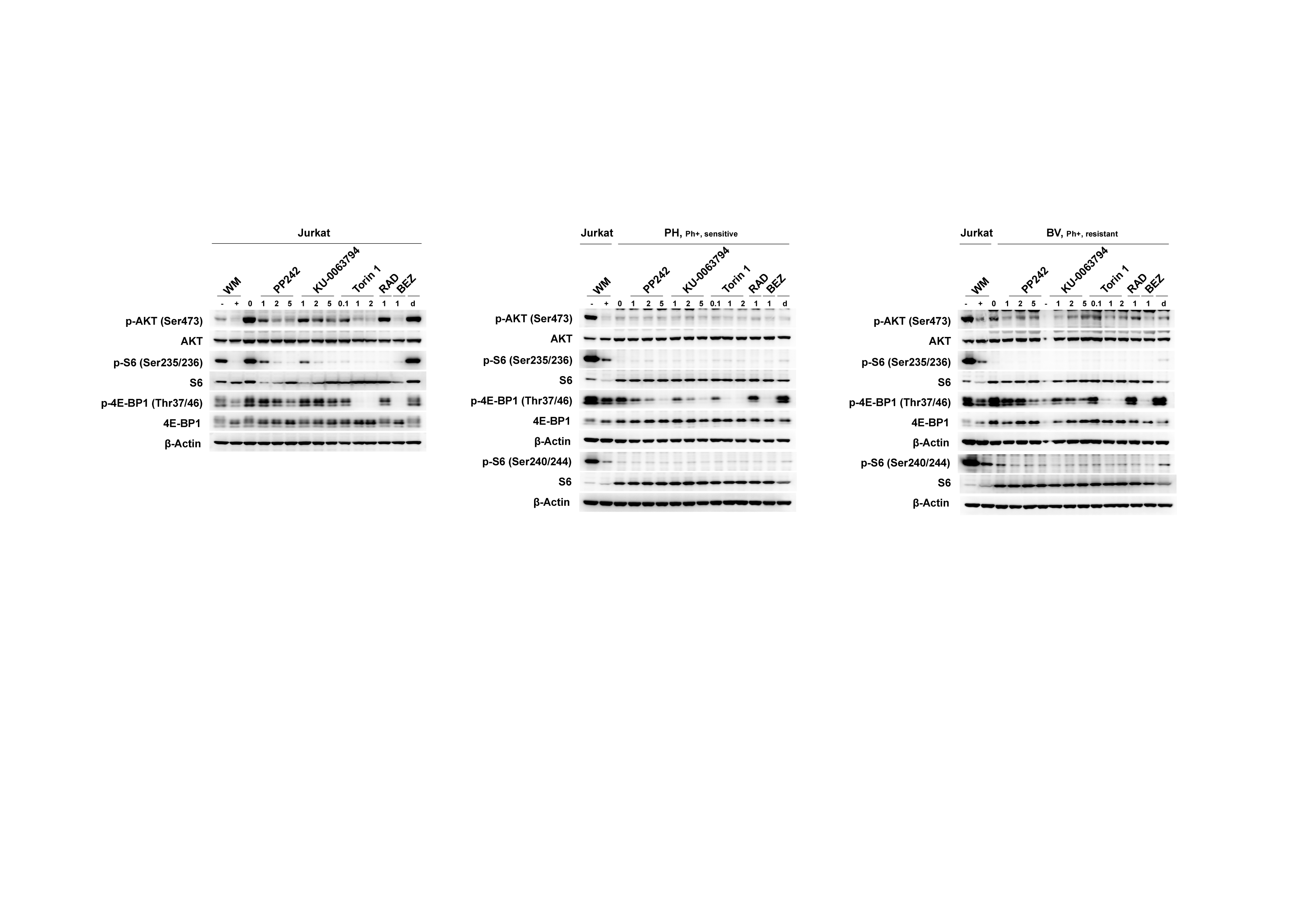

Supplement: Figure S2 — The impact of combined mTORC1 and mTORC2 inhibition in B-ALL on AKT, S6 and 4E-BP1 phosphorylation. BCR-ABL+ (PH, BV) and Jurkat cells were treated with increasing concentrations of KU-0063794, PP242, Torin 1 for 2h. Lysates of these cells were used for the detection of phosphorylated and total AKT, S6 and 4E-BP1 by Western blotting. Lysates of untreated Jurkat cells were used as positive controls and those of cells treated for 2h with 1µM Wortmannin (WM) served as negative controls. β-Actin was used as loading control. d = DMSO control. (TIF) [file pone.0080070.s002.tif]
